# Anti-Kekulé number of the {(3, 4), 4}-fullerene^*^

**DOI:** 10.3389/fchem.2023.1132587

**Published:** 2023-02-24

**Authors:** Rui Yang, Huimin Jia

**Affiliations:** School of Mathematics and Information Science, Henan Polytechnic University, Jiaozuo, Henan, China

**Keywords:** anti-Kekulé set, anti-Kekulé number, {(3,4),4}-fullerene, perfect matching, matching

## Abstract

A *{(3,4),4}-fullerene graph* *G* is a 4-regular plane graph with exactly eight triangular faces and other quadrangular faces. An edge subset *S* of *G* is called an *anti-Kekulé set*, if *G* − *S* is a connected subgraph without perfect matchings. The *anti-Kekulé number* of *G* is the smallest cardinality of anti-Kekulé sets and is denoted by $ak\left(G\right)$ . In this paper, we show that $4\leq ak\left(G\right)\leq 5$ ; at the same time, we determine that the {(3, 4), 4}-fullerene graph with anti-Kekulé number 4 consists of two kinds of graphs: one of which is the graph $H_{1}$ consisting of the tubular graph $Q_{n}\;\left(n\geq 0\right)$ , where *Q*_*n*_ is composed of $n\;\left(n\geq 0\right)$ concentric layers of quadrangles, capped on each end by a cap formed by four triangles which share a common vertex (see Figure 2 for the graph *Q*_*n*_); and the other is the graph $H_{2}$ , which contains four diamonds *D*_1_, *D*_2_, *D*_3_, and *D*_4_, where each diamond $D_{i}\;\left(1\leq i\leq 4\right)$ consists of two adjacent triangles with a common edge $e_{i}\;\left(1\leq i\leq 4\right)$ such that four edges *e*_1_, *e*_2_, *e*_3_, and *e*_4_ form a matching (see Figure 7D for the four diamonds *D*_1_ − *D*_4_). As a consequence, we prove that if $G\in H_{1}$ , then $ak\left(G\right)=4$ ; moreover, if $G\in H_{2}$ , we give the condition to judge that the anti-Kekulé number of graph *G* is 4 or 5.

## 1 Introduction

A *{(3,4),4}-fullerene graph* *G* is a 4-regular plane graph with exactly eight triangular faces and other quadrangular faces. This concept of the {(3, 4), 4}-fullerene comes from Deza’s *{(R,k)}-fullerene* (Deza and Sikirić, 2012). Fixing *R* ⊂ *N*, a {(*R*, *k*)}-fullerene graph is a *k*-regular $\left(k\geq 3\right)$ , and it is mapped on a sphere whose faces are i-gons $\left(i\in R\right)$ . A *{(a,b),k}-fullerene* is {(*R*, *k*)}-fullerene with $R=\left\{a,b\right\}\;\left(1\leq a\leq b\right)$ . The {(*a*, *b*), *k*}-fullerene draws attention because it includes the mostly widely researched graphs, such as fullerenes (i.e.,{(5, 6), 3}-fullerenes), boron–nitrogen fullerenes (i.e.,{(4, 6), 3}-fullerenes), and (3,6)-fullerenes (i.e.,{(3, 6), 3}-fullerenes) (Yang and Zhang, 2012).

The anti-Kekulé number of a graph was introduced by Vukičević and Trinajstić (2007). They introduced the anti-Kekulé number as the smallest number of edges that have to be removed from a benzenoid to remain connected but without a Kekulé structure. Here, a Kekulé structure corresponds to a perfect matching in mathematics; it is known that benzenoid hydrocarbon has better stability if it has a lower anti-Kekulé number. Veljan and Vukičević (2008) found that the anti-Kekulé numbers of the infinite triangular, rectangular, and hexagonal grids are 9, 6 and 4, respectively. Zhang et al. (2011) proved that the anti-Kekulé number of cata-condensed phenylenes is 3. For fullerenes, Vukičević (2007) proved that *C*_60_ has anti-Kekulé number 4, and Kutnar et al. (2009) showed that the leapfrog fullerenes have the anti-Kekulé number 3 or 4 and that for each leapfrog fullerene, the anti-Kekulé number can be established by observing the finite number of cases independent of the size of the fullerene. Furthermore, this result was improved by Yang et al. (2012) by proving that all fullerenes have anti-Kekulé number 4.

In general, Li et al. (2019) showed that the anti-Kekulé number of a 2-connected cubic graph is either 3 or 4; moreover, all (4,6)-fullerenes have the anti-Kekulé number 4, and all the (3,6)-fullerenes have anti-Kekulé number 3. Zhao and Zhang (2020) confirmed all (4,5,6)-fullerenes have anti-Kekulé number 3, which consist of four sporadic (4,5,6)-fullerenes (*F*_12_, *F*_14_, *F*_18_, and *F*_20_) and three classes of (4,5,6)-fullerenes with at least two and at most six pentagons.

Here, we consider the {(3, 4), 4}-fullerene graphs. In the next section, we recall some concepts and results needed for our discussion. In Section 3, by using Tutte’s Theorem on perfect matching of graphs, we determine the scope of the anti-Kekulé number of the {(3, 4), 4}-fullerene. Finally, we show that the {(3, 4), 4}-fullerene with anti-Kekulé number 4 consists of two kinds of graphs $H_{1},H_{2}$ . As a consequence, we prove that if $G\in H_{1}$ , then $ak\left(G\right)=4$ . Moreover, if $G\in H_{2}$ , we give the condition to judge that the anti-Kekulé number of graph *G* is 4 or 5.

## 2 Definitions and preliminary results

Let $G=\left(V,E\right)$ be a simple and connected plane graph with vertex set *V*(*G*) and edge set *E*(*G*). For $V^{\prime }\subseteq V\left(G\right)$ , *G* − *V*′ denotes the subgraph obtained from *G* by deleting the vertices in *V*′ together with their incident edges. If *V*′ = *v*, we write *G* − *v*. Similarly, for $E^{\prime }\subseteq E\left(G\right)$ , *G* − *E*′ denotes the graph with vertex set *V*(*G*) and edge set $E\left(G\right)-E^{\prime }$ . If *E*′ = *e*, we write *G* − *e*. Let *V*′ be a non-empty set; $G\left[V^{\prime }\right]$ denotes the induced subgraph of *G* induced by the vertices of *V*′; similarly, if $E^{\prime }\subseteq E\left(G\right)$ , $G\left[E^{\prime }\right]$ denotes the induced subgraph of *G* induced by the edges of *E*′.

For a subgraph *H* of *G*, the induced subgraph of *G* induced by vertices of $V\left(G\right)-V\left(H\right)$ is denoted by $\bar{H}$ . A plane graph *G* partitions the rest of the plane into a number of arcwise-connected open sets. These sets are called the *faces* of *G*. A face is said to be *incident* with the vertices and edges in its boundary, and two faces are *adjacent* if their boundaries have an edge in common. Let $F\left(G\right)$ be the set of the faces of *G*.

An *edge-cut* of a connected plane graph *G* is a subset of edges $C\subseteq E\left(G\right)$ such that *G* − *C* is disconnected. A *k* *-edge-cut* is an edge-cut with *k* edges. A graph *G* is *k* *-edge-connected* if *G* cannot be separated into at least two components by removing less than *k* edges. An edge-cut *C* of a graph *G* is c*yclic* if its removal separates two cycles. A graph *G* is *cyclically* *k* *-edge-connected* if *G* cannot be separated into at least two components, each containing a cycle, by removing less than *k* edges. A cycle is called a facial cycle if it is the boundary of a face.

For subgraphs *H*_1_ and *H*_2_ of a plane graph *G*, $E\left(H_{1},H_{2}\right)=E\left(V\left(H_{1}\right),V\left(H_{2}\right)\right)$ represents the set of edges whose two end vertices are in $V\left(H_{1}\right)$ and $V\left(H_{2}\right)$ separately. If $V\left(H_{1}\right)$ and $V\left(H_{2}\right)$ are two non-empty disjoint vertex subsets such that $V\left(H_{1}\right)\cup V\left(H_{2}\right)=V\left(G\right)$ , then $E\left(H_{1},H_{2}\right)$ is an edge-cut of *G*, and we simply write $\nabla \left(H_{1}\right)=\nabla \left(V\left(H_{1}\right)\right)$ or $\nabla \left(H_{2}\right)=\nabla \left(V\left(H_{2}\right)\right)$ . We use $\partial \left(G\right)$ to denote the *boundary* of *G*, that is, the boundary of the infinite face of *G*.

A *matching* *M* of a graph *G* is a set of edges of *G* such that no two edges from *M* have a vertex in common. A matching *M* is *perfect* if it covers every vertex of *G*. A *perfect matching* is also called a Kekulé structure in chemistry.

Let *G* be a connected graph with at least one perfect matching. For $S\subseteq E\left(G\right)$ , we call *S* an *anti-Kekulé* set if *G* − *S* is connected but has no perfect matchings. The smallest cardinality of anti-Kekulé sets of *G* is called the *anti-Kekulé number* and denoted by $ak\left(G\right)$ .

For the edge connectivity of the {(3, 4), 4}-fullerene, we have the following results.

Lemma 2.1 ((Yang et al., 2023) *Lemma 2.3*) *Every* {(3, 4), 4}*-fullerene is cyclically 4-edge-connected.*

Lemma 2.2 ((Yang et al., 2023) *Corollary 2.4*) *Every* {(3, 4), 4}*-fullerene is 4-edge-connected.*

*Q*_*n*_ is the graph consisting of *n* concentric layers of quadrangles, capped on each end by a cap formed by four triangles which share a common vertex as shown in Figure 2. In particular, *Q*_0_ is what we call an octahedron (see Figure 5F).

Lemma 2.3 ((Yang et al., 2023) *Lemma 2.5*) *If* *G* *has a cyclical 4-edge-cut* $E=\left\{e_{1},e_{2},e_{3},e_{4}\right\}$ *, then* $G≅Q_{n}\;\left(n\geq 1\right)$ *, where the four edges* *e*_1_ *,* *e*_2_ *,* *e*_3_ *,* and *e*_4_ *form a matching, and each* *e*_*i*_ *belongs to the intersection of two quadrilateral faces for* *i* = 1, 2, 3, 4*.*

Tutte’s theorem plays an important role in the process of proof.

Theorem 2.4 (Lovász and Plummer, 2009) *(Tutte’s theorem) A graph* *G* *has a perfect matching if and only if for any* $X\subseteq V\left(G\right)$ *,* $o\left(G-X\right)\leq \left|X\right|$ *, where* $o\left(G-X\right)$ *denotes the number of odd components of* *G* − *X* *.*

Here, an odd component of *G* − *X* is *trivial* if it is just a single vertex and *non-trivial* otherwise.

All graph-theoretical terms and concepts used but unexplained in this article are standard and can be found in many textbooks, such as Lovász and Plummer (2009).

## 3 Main results

From now on, let *G* always be a {(3, 4), 4}-fullerene; we called a 4-edge-cut *E* in *G* *trivial* if $E=\nabla \left(v\right)$ , that is, *E* consists of the four edges incident to *v*. By Lemma 2.3, if *E* is a cyclical 4-edge-cut, then the four edges in *E* form a matching. Moreover, if *E* is not a cyclical 4-edge-cut, then *E* is trivial. So, we have the following lemma.

Lemma 3.1 *Let* *G* *be a* {(3, 4), 4}*-fullerene,* $E=\left\{e_{1},e_{2},e_{3},e_{4}\right\}$ *be an 4-edge-cut, but it is not cyclical, then* *E* *is trivial.*

Proof Since $E=\left\{e_{1},e_{2},e_{3},e_{4}\right\}$ is an 4-edge-cut, *G* − *E* is not connected. Then, *G* − *E* has at least two components. Moreover, as *G* is 4-edge-connected by Lemma 2.2, *G* − *E* has at most two components. So, *G* − *E* has exactly two components.

Let *G*_1_, *G*_2_ be two components of *G* − *E*. Since *E* is not cyclical, without loss of generality, we suppose that *G*_1_ is a forest; then, we have

n−e=l, (1)

where *n*, *e*, *l* is the number of vertices, edges, and trees in *G*_1_, respectively. Furthermore, since each vertex of *G* is of degree 4, we have

4n−4=2e. (2)

Combing with equalities 1) and 2), we know *n* = *l* = 1 and *e* = 0, which means *G*_1_ only consists of a single vertex. So, *E* is trivial. □

Lemma 3.1 plays an important role in the proof of the following theorem. Next, we explore the scope of the anti-Kekulé number of {(3, 4), 4}-fullerene.

Theorem 3.2 *Let* *G* *be a* {(3, 4), 4}*-fullerene, then* $4\leq ak\left(G\right)\leq 5$ *.*

**FIGURE 1:**
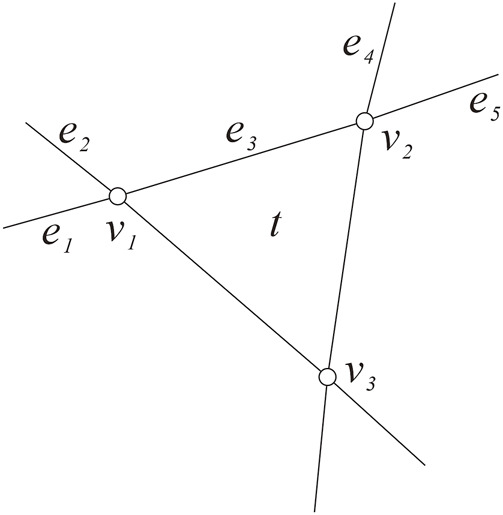
Edges *e*_1_ *e*_2_, *e*_3_, *e*_4_, and *e*_5_.

Proof First, we show $ak\left(G\right)\leq 5$ . Let *t* be any triangle in *G* and the boundary of *t* was labeled *v*_1_ *v*_2_ *v*_3_ along the clockwise direction. Denote the other two edges incident to $v_{1}\;\left(v_{2}\right)$ by $e_{1},e_{2}\;\left(e_{4},e_{5}\right)$ , set *e*_3_ = *v*_1_ *v*_2_, then *e*_1_, *e*_2_, *e*_3_, *e*_4_, and *e*_5_ are pairwise different, set $E^{\prime }=\left\{e_{1},e_{2},e_{3},e_{4},e_{5}\right\}$ (see Figure 1) and *G*′ = *G* − *E*′.

In order to show $ak\left(G\right)\leq 5$ , we only need to prove that *G*′ is connected and has no perfect matchings. Then, *G*′ has no perfect matchings since the two edges *v*_1_ *v*_3_, *v*_2_ *v*_3_ cannot be covered by a perfect matching at the same time in *G*′.

In the following, we show that *G*′ is connected. We proved this using reduction to absurdity, suppose *G*′ is not connected, then *G*′ has a component (say *G*_1_) containing vertices *v*_1_, *v*_2_, and *v*_3_, as *v*_1_, *v*_2_, and *v*_3_ are connected by the path *v*_1_ *v*_3_ *v*_2_ in *G*_1_. On the other hand, since *e*_3_ = *v*_1_ *v*_2_ connects two vertices *v*_1_, *v*_2_ in *G* and $E^{\prime }=\left\{e_{1},e_{2},e_{3},e_{4},e_{5}\right\}$ is an edge cut of *G*, even if we remove five edges, *e*_1_, *e*_2_, *e*_3_, *e*_4_, and *e*_5_, to disconnect *G*, it is actually the same as removing four edges, *e*_1_, *e*_2_, *e*_4_, and *e*_5_ (see Figure 1); that is, $E_{1}=\left\{e_{1},e_{2},e_{4},e_{5}\right\}$ is an 4-edge-cut. Moreover, due to Lemma 2.3, *E*_1_ cannot be a cyclical 4-edge-cut as *e*_1_, *e*_2_, *e*_4_, and *e*_5_ is not a matching. Then, according to Lemma 3.1, *E*_1_ is a trivial 4-edge-cut. Thus, *G*_1_ or $\bar{G_{1}}$ is a single vertex, both of which are impossible by the definition of *G*. So *G*′ is connected. Thus,

$$ak\left(G\right)\leq 5.$$ (3)

Finally, we show $ak\left(G\right)\geq 4$ . By the definition of an anti-Kekulé set, suppose $E_{1}^{\prime }=\left\{e_{1}^{\prime },e_{2}^{\prime },e_{3}^{\prime },\ldots ,e_{k}^{\prime }\right\}$ was the smallest anti-Kekulé set of *G*, that is, $ak\left(G\right)=k$ . Then, $G_{1}^{\prime }=G-E_{1}^{\prime }$ was connected and has no perfect matching. Hence, according to Theorem 2.4, there exists a non-empty subset $X_{0}\subseteq V\left(G_{1}^{\prime }\right)$ such that $o\left(G_{1}^{\prime }-X_{0}\right)>\left|X_{0}\right|$ , since $\left|V\left(G_{1}^{\prime }\right)\right|=\left|V\left(G\right)\right|$ and $\left|V\left(G\right)\right|$ is even, $o\left(G_{1}^{\prime }-X_{0}\right)$ and $\left|X_{0}\right|$ have the same parity. Consequently,

$$o\left(G_{1}^{\prime }-X_{0}\right)\geq \left|X_{0}\right|+2$$ (4)

For the sake of convenience, we let $\alpha =o\left(G_{1}^{\prime }-X_{0}\right)$ . If we chose an *X*_0_ with the maximum size, then $G_{1}^{\prime }-X_{0}$ has no even components. On the contrary, we suppose there exists an even component (say *F*) of $G_{1}^{\prime }-X_{0}$ . For any vertex $v\in V\left(F\right)$ , $o\left(F-v\right)\geq 1$ . Let $X^{\prime }=X_{0}\cup \left\{v\right\}$ , thus $o\left(G_{1}^{\prime }-X^{\prime }\right)=o\left(G_{1}^{\prime }-X_{0}\right)+o\left(F-v\right)\geq \left|X_{0}\right|+2+1=\left|X^{\prime }\right|+2$ , which is a contradiction to the choice of *X*_0_.

In addition, $E_{1}^{\prime }$ is the smallest anti-Kekulé set of *G*, then $G_{1}^{\prime }+e_{i}^{\prime }$ has perfect matchings for any edge $e_{i}^{\prime }\in E_{1}^{\prime }$ for 1 ≤ *i* ≤ *k*. On the other hand, the number of odd components of $G_{1}^{\prime }-X_{0}$ was not decreased or decreased by at most one or two if we add one edge $e_{i}^{\prime }$ to $G_{1}^{\prime }$ , that is,

$$\left|X_{0}\right|\geq o\left(G_{1}^{\prime }+e_{i}^{\prime }-X_{0}\right)\geq \alpha ‐2.$$ (5)

By inequality (4), we have

$$\left|X_{0}\right|\leq \alpha ‐2.$$ (6)

Combined with inequalities (5) and (6), we have $\alpha =\left|X_{0}\right|+2$ and each edge $e_{i}^{\prime }\in E_{1}^{\prime }$ connects two odd components of $G_{1}^{\prime }-X_{0}$ . Let *H*_1_, *H*_2_, *H*_3_, …, *H*_*α*_ be the odd components of $G_{1}^{\prime }-X_{0}$ . Then, due to Lemma 2.2, $\left|\nabla \left(H_{i}\right)\right|\geq 4\;\left(1\leq i\leq \alpha \right)$ ; therefore,

$$4\alpha -2k\leq \overset{\alpha }{\underset{i=1}{\sum }}\left|\nabla \left(H_{i}\right)\right|-2\left|E_{1}^{\prime }\right|\leq 4\left|X_{0}\right|=4\left(\alpha -2\right).$$ (7)

Thus, *k* ≥ 4, that is, $ak\left(G\right)\geq 4$ . We know that $4\leq ak\left(G\right)\leq 5$ .

**FIGURE 2:**
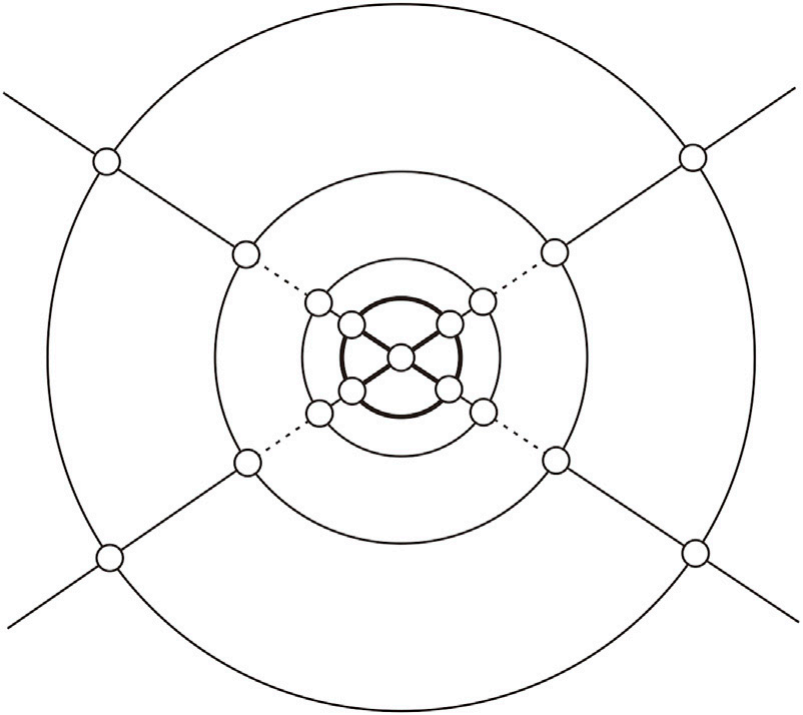
{(3,4),4}-Fullerene *Q*_*n*_, where the bold segments indicate the cap of *Q*_*n*_ (*n* ≥0).

By Theorem 3.2, we know that $4\leq ak\left(G\right)\leq 5$ . Next, we give the characterization of {(3, 4), 4}-fullerenes with anti-Kekulé number 4. Before, we define $H_{1}=\left\{Q_{n}\left|n\geq 0\right.\right\}$ , where *Q*_*n*_ is shown in Figure 2. The structure of two adjacent triangles is called a *diamond*. In a diamond, the common edge of the two triangles is called the *diagonal edge*. The subgraph consisting of four diamonds such that the four diagonal edges form a matching is denoted by *D*, that is, $D=\overset{4}{\underset{i=1}{⋃}}D_{i}$ (see Figure 7D for the four diamonds *D*_1_ − *D*_4_). Let $H_{2}=\left\{G|D\subseteq G\right\}$ . So, we have the following theorem.

Theorem 3.3 *Let* *G* *be a* {(3, 4), 4}*-fullerene, if* $ak\left(G\right)=4$ *, then* $G\in H_{1}$ *or* $G\in H_{2}$ *.*

Proof Let *E*_0_ be the anti-Kekulé set of *G* such that $\left|E_{0}\right|=4$ , set *G*_0_ = *G* − *E*_0_. Then, *G*_0_ is connected without perfecting matchings. Thus, by Theorem 2.4, there exists a non-empty subset $X_{0}\subseteq V\left(G_{0}\right)$ such that $o\left(G_{0}-X_{0}\right)>\left|X_{0}\right|$ . For convenience, let $\alpha =o\left(G_{0}-X_{0}\right)$ , since *α* and $\left|X_{0}\right|$ have the same parity, that is,

$$\alpha \geq \left|X_{0}\right|+2.$$ (8)

We choose an *X*_0_ satisfying Ineq. (8) with the maximum size. Then, a proof similar to the proof of Theorem 3.2 is used to prove $ak\left(G\right)\geq 4$ . We can know *G*_0_ − *X*_0_ has no even components. Let *H*_1_, *H*_2_, *H*_3_, …, *H*_*α*_ be all the odd components of *G*_0_ − *X*_0_, set $H=\overset{\alpha }{\underset{i=1}{\cup }}H_{i}$ .

**FIGURE 3:**
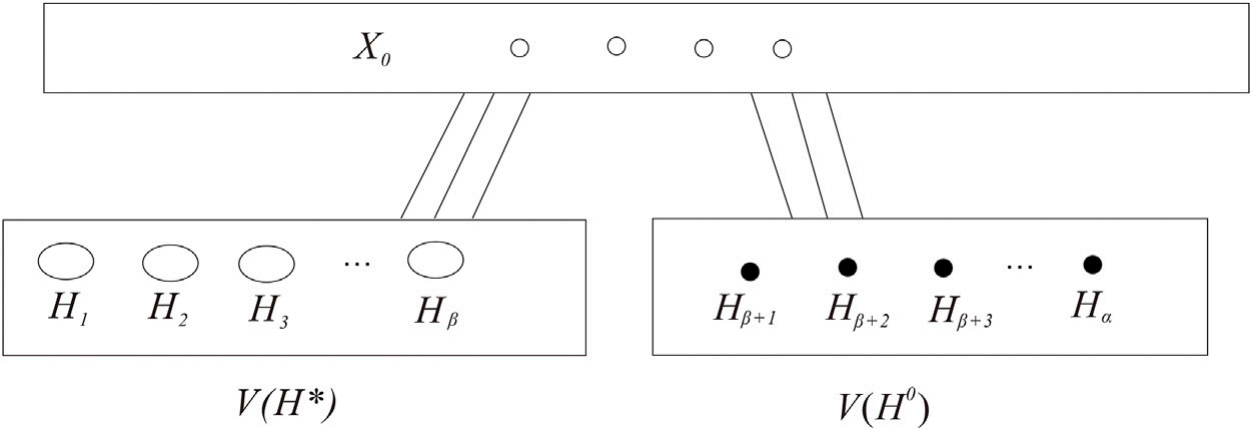
$V\left(G\right)$ is divided into *X*_0_, $V\left(H^{*}\right)$ , and $V\left(H^{0}\right)$ .

Let *H*_1_, *H*_2_, *H*_3_, …, *H*_*β*_ be the non-trivial odd components of *G*_0_ − *X*_0_, set $H^{*}=\overset{\beta }{\underset{i=1}{\cup }}H_{i}$ . Let *H*_*β*+1_, *H*_*β*+2_, *H*_*β*+3_, …, *H*_*α*_ be the trivial odd components of *G*_0_ − *X*_0_, set $H^{0}=\overset{\alpha }{\underset{i=\beta +1}{\cup }}H_{i}$ . Then, $V\left(G\right)$ is divided into *X*_0_, $V\left(H^{*}\right)$ , $V\left(H^{0}\right)$ (see Figure 3 the partition of $V\left(G\right)$ ).

Since $ak\left(G\right)=4$ , all equalities in Ineq. (7) of Theorem 3.2 hold. The first equality in Ineq. (7) holds if and only if $\left|\nabla \left(H_{i}\right)\right|=4\left(1\leq i\leq \alpha \right)$ , and the second equality in Ineq. 7) holds if and only if there is no edge in the subgraph $G_{0}\left[X_{0}\right]$ ; that is, *X*_0_ is an independent set of *G*_0_. Moreover, each edge of *E*_0_ connects two components in *H* and $\left|X_{0}\right|=\alpha -2$ . Since $\left|\nabla \left(H_{j}\right)\right|=4\;\left(1\leq j\leq \alpha \right)$ , $\nabla \left(H_{j}\right)$ is a cyclical 4-edge-cut of *G* or not.

Next, we distinguish the following two cases to complete the proof of Theorem 3.3.

**Case 1:** There exists one *H*_*j*_ such that $\nabla \left(H_{j}\right)$ is a cyclical 4-edge-cut.

**FIGURE 4:**
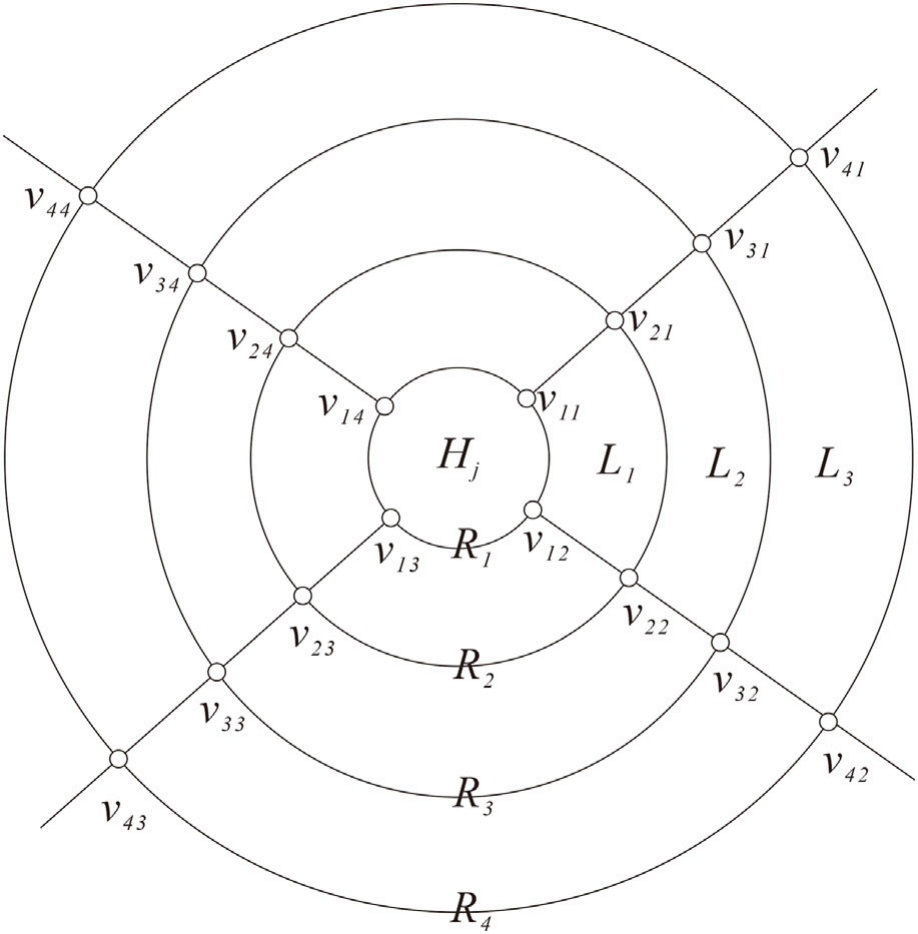
Labeling of $G\left[V\left(\bar{H_{j}}\cup \partial \left(H_{j}\right)\right)\right]$ .

By Lemma 2.3, $G≅Q_{n}\;\left(n\geq 1\right)$ , which means the four edges of $\nabla \left(H_{j}\right)$ form a matching. Without loss of generality, we supposed *H*_*j*_ consists of *s* layers of quadrangular faces and the cap of *H*_*j*_ is entirely in the interior of the boundary cycle $\partial \left(H_{j}\right)$ . Then, $G\left[V\left(\bar{H_{j}}\cup \partial \left(H_{j}\right)\right)\right]$ induced by the vertices of $\bar{H_{j}}$ and the boundary of $\partial \left(H_{j}\right)$ consists of *n* − *s* layers of quadrangular faces and a cap, for convenience, set *m* = *n* − *s*, let *L*_1_, *L*_2_, *L*_3_, …, *L*_*m*_ be all the layers and *C* be the cap of $G\left[V\left(\bar{H_{j}}\cup \partial \left(H_{j}\right)\right)\right]$ , where quadrangular layer *L*_*i*_ is adjacent to *L*_*i*−1_ and *L*_*i*+1_ for 2 ≤ *i* ≤ *m* − 1, *L*_1_ is adjacent to *H*_*j*_, and *L*_*m*_ is adjacent to *C*. Set *R*_1_ = *H*_*j*_ ∩ *L*_1_ and *R*_*m*+1_ = *C* ∩ *L*_*m*_. For 2 ≤ *i* ≤ *m*, let *R*_*i*_ = *L*_*i*−1_ ∩ *L*_*i*_. The vertices on $R_{i}\;\left(i=1,2,3,\ldots ,m+1\right)$ are recorded as *v*_*i*1_, *v*_*i*2_, *v*_*i*3_, and *v*_*i*4_ (*i* = 1, 2, 3, …, *m* + 1) in a clockwise direction and *v*_*i*1_, *v*_*i*3_, and *v*_*i*2_, *v*_*i*4_, are on the same line, respectively (see Figure 4). Since $\nabla \left(H_{j}\right)$ is a cyclical 4-edge-cut, set $\nabla \left(H_{j}\right)=\left\{e_{1}^{\prime },e_{2}^{\prime },e_{3}^{\prime },e_{4}^{\prime }\right\}$ . Without loss of generality, set $e_{i}^{\prime }=v_{1i}v_{2i}\;\left(1\leq i\leq 4\right)$ . The vertices shared by the four triangles on the two caps are represented by *v*′, *v* ^″^, respectively, such that *v*′ is in *H*_*j*_ and *v* ^″^ is in $\bar{H_{j}}$ .

Next, we analyze whether the edges of $\nabla \left(H_{j}\right)$ belongs to *E*_0_ or not, which is divided into the following five subcases.

**Subcase 1.1:** All the edges of $\nabla \left(H_{j}\right)$ belong *E*_0_.

That is, $e_{i}^{\prime }\in E_{0}$ for all *i* = 1, 2, 3, 4. Since each edge of *E*_0_ connects two components of *H* and there are four edges $e_{1}^{\prime },e_{2}^{\prime },e_{3}^{\prime },e_{4}^{\prime }$ belonging to *E*_0_. All the vertices of $\bar{H_{j}}$ belong to $V\left(H^{*}\right)$ , which means *X*_0_ = ∅, a contradiction.

**Subcase 1.2:** Exactly three edges of $\nabla \left(H_{j}\right)$ belong to *E*_0_.

Without loss of generality, suppose $e_{1}^{\prime },e_{2}^{\prime },e_{3}^{\prime }\in E_{0}$ , then *v*_24_ ∈ *X*_0_ and $v_{21},v_{22},v_{23}\in V\left(H\right)$ , that is, *v*_21_, *v*_22_, *v*_23_ belong to $V\left(H^{*}\right)$ or $V\left(H^{0}\right)$ .

If all of *v*_21_, *v*_22_, and *v*_23_ belong to $V\left(H^{0}\right)$ , then *v*_21_ *v*_22_, *v*_22_ *v*_23_ ∈ *E*_0_, immediately $\left|E_{0}\right|>4$ , which contradicts $\left|E_{0}\right|=4$ . This contradiction means at least one of *v*_21_, *v*_22_, and *v*_23_ belongs to $V\left(H^{*}\right)$ (say $V\left(H_{1}\right)$ ), then by Lemma 2.3 and Lemma 3.1, either $\nabla \left(H_{1}\right)$ is a cyclical 4-edge-cut and the four edges in $\nabla \left(H_{1}\right)$ form a matching or $\nabla \left(H_{1}\right)$ is trivial. However, since *H*_1_ is a non-trivial odd component of *G*_0_ − *X*_0_, $\left|V\left(H_{1}\right)\right|\geq 3$ . Thus, $\nabla \left(H_{1}\right)$ is not a trivial 4-edge-cut. That is, $\nabla \left(H_{1}\right)$ is a cyclical 4-edge-cut, and the four edges in $\nabla \left(H_{1}\right)$ form a matching. Now, if *v*_21_ (or *v*_23_) belong to $V\left(H_{1}\right)$ , then *v*_21_ *v*_24_, *v*_21_ *v*_11_ (or *v*_23_ *v*_24_, *v*_23_ *v*_13_) belong to $\nabla \left(H_{1}\right)$ , but they do not form a matching, a contradiction. Thus, both $v_{21},v_{23}\in V\left(H^{0}\right)$ and $v_{22}\in V\left(H_{1}\right)$ . Immediately, we have *v*_21_ *v*_22_, *v*_22_ *v*_23_ ∈ *E*_0_ and $\left|E_{0}\right|>4$ , which contradicts $\left|E_{0}\right|=4$ . This contradiction means there cannot be three edges of $\nabla \left(H_{j}\right)$ belonging to *E*_0_.

**Subcase 1.3:** Exactly two edges of $\nabla \left(H_{j}\right)$ belong to *E*_0_.

Then, by symmetry, $e_{1}^{\prime },e_{2}^{\prime }\in E_{0}$ or $e_{1}^{\prime },e_{3}^{\prime }\in E_{0}$ .

First, if $e_{1}^{\prime },e_{2}^{\prime }\in E_{0}$ , then *v*_23_, *v*_24_ ∈ *X*_0_ and $v_{23}v_{24}\in E\left(X_{0}\right)$ , which contradicts that $E\left(X_{0}\right)=\emptyset$ .

**Claim 1:** For a quadrangular face *q* with $\partial \left(q\right)=abcda$ with clock direction such that $a\in X_{0},b\in V\left(H^{0}\right)$ , then $c,d\in V\left(H^{0}\right)$ or $c,d\in V\left(H^{*}\right)$ or $c\in X_{0},d\in V\left(H^{0}\right)$ .

Proof Since $E\left(X_{0}\right)=\emptyset$ , $d\in V\left(H^{0}\right)\cup V\left(H^{*}\right)$ . If $d\in V\left(H^{0}\right)$ , then $c\notin V\left(H^{*}\right)$ by Lemma 2.3 and Lemma 3.1, thus *c* ∈ *X*_0_ or $c\in V\left(H^{0}\right)$ .

If $d\in V\left(H^{*}\right)$ , then also by Lemma 2.3 and Lemma 3.1, we can know $c\in V\left(H^{*}\right)$ and the claim holds.

By Claim 1, next, if $e_{1}^{\prime },e_{3}^{\prime }\in E_{0}$ , then $v_{22},v_{24}\in V\left(X_{0}\right)$ , $v_{21},v_{23}\in V\left(H\right)$ . If all the vertices of *v*_21_, *v*_22_, *v*_23_, and *v*_24_ belong to the cap of $\bar{H_{j}}$ , that is, all of *v*_21_, *v*_22_, *v*_23_, and *v*_24_ are adjacent to *v* ^″^, then as $\left|E_{0}\right|=4$ and $e_{1}^{\prime },e_{3}^{\prime }\in E_{0}$ , we can know *v* ^″^ ∈ *H* ^0^ and *v*_21_ *v* ^″^, *v*_23_ *v* ^″^ ∈ *E*_0_, and we have the {(3, 4), 4}-fullerenes *Q*_*s*+1_, that is, *m* = 1.

If all the vertices of *v*_21_, *v*_22_, *v*_23_, and *v*_24_ do not belong to the cap of $\bar{H_{j}}$ , that is, the layer *L*_2_ consists of four quadrangular faces, then, for the quadrangular face $q\in F\left(L_{2}\right)$ , the vertices on $\partial \left(q\right)$ belong to *X*_0_, *H* ^0^, *H* ^0^, *H* ^0^ or *X*_0_, *H* ^0^, H*, H* or *X*_0_, *H* ^0^, *X*_0_, *H* ^0^ by Claim 1.

If the former case holds, that is, there exists one face $q\in F\left(L_{2}\right)$ such that the boundary of *q* is of the form *X*_0_, *H* ^0^, *H* ^0^, and *H* ^0^, then immediately we can have $\left|E_{0}\right|>4$ , a contradiction.

If the second case holds, that is, there exists one face $q\in F\left(L_{2}\right)$ such that the boundary of *q* is of the form *X*_0_, *H* ^0^, H*, and H*, then by Claim 1 and since $\left|E_{0}\right|=4$ , we can know all the faces of *L*_2_ are of the form *X*_0_, *H* ^0^, H*, and H*, that is, all the vertices of $\bar{H_{j}\cup L_{1}}$ belong to $V\left(H^{*}\right)$ . In this case, we also have $G\in H_{1}$ .

By the aforementioned discussion and Claim 1, next, we suppose all the quadrangular faces of *L*_2_ are of the form *X*_0_, *H* ^0^, *X*_0_, and *H* ^0^. Then, we can use the aforementioned same analysis to the layer *L*_3_ as *L*_2_, since *G ≅ Q*_*n*_ and *H*_*j*_ consists of *s* layers of quadrangular faces; after finite steps (say *t* steps), we obtain *t* layers *L*_2_, *L*_3_, …, *L*_*t*+1_ such that all the faces of $L_{i}\left(2\leq i\leq t+1\right)$ are of form *X*_0_, *H* ^0^, *X*_0_, and *H* ^0^ and either the four vertices on $\partial \left(R_{t+2}\right)$ are adjacent to $v^{\prime \prime }\;\left(v^{\prime \prime }\in V\left(H^{0}\right)\right)$ or all the vertices of $\bar{H_{j}\cup L_{1}\cup L_{2}\cup \cdots \cup L_{t+1}}$ belong to $V\left(H^{*}\right)$ .

If the four vertices on $\partial \left(R_{t+2}\right)$ are adjacent to $v^{\prime \prime }\;\left(v^{\prime \prime }\in V\left(H^{0}\right)\right)$ , then *m* = *t* + 1, *n* = *s* + *t* + 1 and $G\in H_{1}$ . If all the vertices of $\bar{H_{j}\cup L_{1}\cup L_{2}\cup \cdots \cup L_{t+1}}$ belong to $V\left(H^{*}\right)$ (say $V\left(H_{1}\right)$ ), suppose *H*_1_ consists of *p* layers of quadrangular faces, then *m* = *t* + *p* + 2, *n* = *s* + *t* + *p* + 2, and also $G\in H_{1}$ .

To sum up, if exactly two edges of $\nabla \left(H_{j}\right)$ belong to *E*_0_, then $G\in H_{1}$ .

**Subcase 1.4:** Exactly one edge of $\nabla \left(H_{j}\right)$ belong to *E*_0_.

Without loss of generality, suppose $e_{1}^{\prime }\in E_{0}$ , then *v*_22_, *v*_23_, *v*_24_ ∈ *X*_0_, $v_{22}v_{23},v_{23}v_{24}\in E\left(X_{0}\right)$ , which contradicts that *X*_0_ is an independent set of *G*_0_.

**Subcase 1.5:** No edge of $\nabla \left(H_{j}\right)$ belongs to *E*_0_.

Thus, $\overset{4}{\underset{i=1}{⋃}}v_{2i}\subseteq X_{0}$ , so $v_{21}v_{22},v_{22}v_{23},v_{23}v_{24},v_{24}v_{21}\in E\left(X_{0}\right)$ , which contradicts $E\left(X_{0}\right)=\phi$ .

**Case 2:** $\nabla \left(H_{j}\right)$ is not a cyclical 4-edge-cut of *G* for all 1 ≤ *j* ≤ *α*.

For convenience, set $E_{0}=\left\{e_{1},e_{2},e_{3},e_{4}\right\}$ . Here, first, we give the idea of proof, then we will show that *G*_0_ = *G* − *E*_0_ is bipartite by proving $\left|V\left(H_{i}\right)\right|=1\;\left(1\leq i\leq \alpha \right)$ . Since *G* has exactly eight triangular faces and $\left|E_{0}\right|=4$ , which implies that each edge *e*_*i*_ of *E*_0_ is the common edge of two triangles, by discussing all possible subgraphs formed by facial cycles containing an edge of *E*_0_, we show that $G\in H_{1}$ or $G\in H_{2}$ .

Since $\nabla \left(H_{j}\right)$ is not a cyclical 4-edge-cut of *G* for all 1 ≤ *j* ≤ *α*, *H*_*j*_ or $\bar{H_{j}}$ is a singleton by Lemma 3.1. Since *X*_0_ is non-empty and $\alpha =\left|X_{0}\right|+2$ , which means *H*_*j*_ is a singleton vertex, that is, $\left|V\left(H_{j}\right)\right|=1\;\left(1\leq j\leq \alpha \right)$ .

Let *Y*_0_ denote the set of all singletons *y*_*i*_ from each $H_{i}\;\left(1\leq i\leq \alpha \right)$ , and denote the vertices of *X*_0_ by $x_{i}\;\left(1\leq i\leq \left|X_{0}\right|\right)$ , so $G_{0}=\left(X_{0},Y_{0}\right)$ is bipartite. For convenience, we color the vertices white in *X*_0_ and black in *Y*_0_.

Next, we consider possible subgraphs of *G* containing all edges of *E*_0_. By the Euler theorem, *G* has exactly eight triangular faces because $G_{0}=\left(X_{0},Y_{0}\right)$ is bipartite; each edge *e*_*i*_ of *E*_0_ is the common edge of two triangles and connects two vertices in *Y*_0_, that is, every edge *e*_*i*_ ∈ *E*_0_ belongs to a diamond, say *D*_*i*_, *i* = 1, 2, 3, 4 and $F\left(D_{i}\right)\cap F\left(D_{j}\right)=\emptyset \;\left(i\neq j,i,\;j=1,2,3,4\right)$ .

**Claim 2:** If $G\left[E_{0}\right]$ has one component, then *G ≅ Q*_0_, where *Q*_0_ is the octahedron.

**FIGURE 5:**
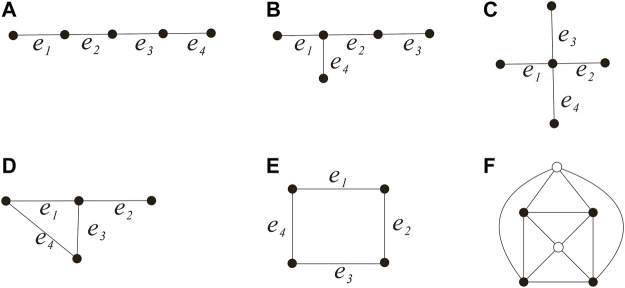
$G\left[E_{0}\right]$ has one component and the {(3,4),4}-fullerene *Q*_0_ **(A–F)**.

Proof If $G\left[E_{0}\right]$ has one component, then we have the subgraphs shown in Figures 5A, B, C) if $G\left[E_{0}\right]$ is a tree and Figures 5D, E if $G\left[E_{0}\right]$ has cycles. If $G\left[E_{0}\right]$ is isomorphism to the graph shown in Figure 5A, then the two diamonds *D*_1_, *D*_2_ are adjacent and they form one cap of *Q*_*n*_. Set *D*_12_ = *D*_1_ ∪ *D*_2_, then $\nabla \left(D_{12}\right)$ forms an 4-edge-cut. On the other hand, by Lemma 2.3 and Lemma 3.1, $\nabla \left(D_{12}\right)$ is a cyclical 4-edge-cut and *G ≅ Q*_*p*_ or $\nabla \left(D_{12}\right)$ is trivial. If $\nabla \left(D_{12}\right)$ is a cyclical 4-edge-cut, then $G≅Q_{p}\left(p\geq 1\right)$ and *e*_3_ belongs to a quadrangular face, which contradicts that the two faces containing *e*_3_ are triangles. If $\nabla \left(D_{12}\right)$ is a trivial 4-edge-cut, that is, $\bar{D_{12}}$ is a singleton, which is impossible as the two vertices of *e*_4_ belong to $V\left(\bar{D_{12}}\right)$ . Thus, $G\left[E_{0}\right]$ cannot be isomorphism to the subgraph shown in Figure 5A. All the situations of Figures 5B–D contradicts $F\left(D_{i}\right)\cap F\left(D_{j}\right)=\emptyset \;\left(i\neq j,i,\;j=1,2,3,4\right)$ .

If $G\left[E_{0}\right]$ is isomorphic to the graph shown in Figure 5E, then in order to guarantee $F\left(D_{i}\right)\cap F\left(D_{j}\right)=\emptyset \;\left(i\neq j,i,\;j=1,2,3,4\right)$ , the four diamonds *D*_1_, *D*_2_, *D*_3_, and *D*_4_ forms two caps of *Q*_*n*_ such that the cycle induced by *E*_0_ is exactly the intersecting of the two caps. Immediately, we have the graph *Q*_0_ (see Figure 5F the octahedron *Q*_0_), that is, *G ≅ Q*_0_ if $G\left[E_{0}\right]$ has one component, so $G\in H_{1}$ .

In accordance with Claim 2, next, we assume that $G\left[E_{0}\right]$ is not connected, so $G\left[E_{0}\right]$ has at least two and at most four components. Then, we have the following three cases.

**Subcase 2.1:** $G\left[E_{0}\right]$ has exactly two components.

**FIGURE 6:**
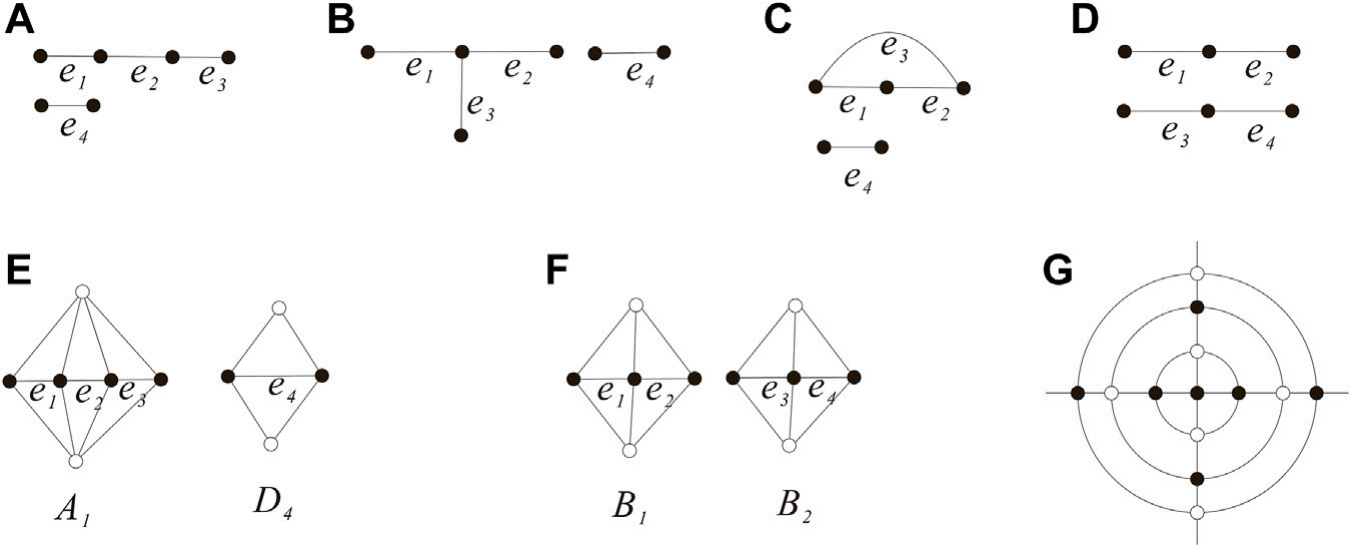
$G\left[E_{0}\right]$ has two components and the {(3,4),4}-fullerenes $Q_{l}\;\left(l\geq 1\right)$ **(A–G)**.

By symmetry, the subgraph induced by *E*_0_ has four cases as shown in Figures 6A–D. Then, the graph *G* which contains the subgraphs shown in Figure 6B contradicts $F\left(D_{i}\right)\cap F\left(D_{j}\right)=\emptyset \;\left(i\neq j,i,\;j=1,2,3,4\right)$ . If *G* contains the subgraph shown in Figure 6C, then the three edges *e*_1_, *e*_2_, and *e*_3_ belong to the same triangular face as every 3-length cycle of a {(3, 4), 4}-fullerene must be the boundary of a triangular face by Lemma 2.2, which contradicts that $F\left(D_{i}\right)\cap F\left(D_{j}\right)=\emptyset \;\left(i\neq j,i,\;j=1,2,3,4\right)$ .

If $G\left[E_{0}\right]$ is isomorphic to the graph as shown in Figure 6A, then the three edges *e*_1_, *e*_2_, and *e*_3_ belong to three diamonds *D*_1_, *D*_2_, and *D*_3_, respectively, and we have the subgraph *A*_1_ consisting of *D*_1_, *D*_2_, and *D*_3_ (see Figure 6E) such that $\left|\nabla \left(A_{1}\right)\right|=2$ and *A*_1_, *D*_4_ are disjoint. By the definition of *G*, we can know the two 3-degree vertices on $\partial \left(A_{1}\right)$ must be adjacent and we obtain *G ≅ Q*_0_, which contradicts that *A*_1_, *D*_4_ are disjoint.

If $G\left[E_{0}\right]$ is isomorphic to the graph as shown in Figure 6D, then *D*_1_, *D*_2_ are adjacent, and *D*_3_, *D*_4_ are adjacent. Set *B*_1_ = *D*_1_ ∪ *D*_2_, *B*_2_ = *D*_3_ ∪ *D*_4_. Since the two edges *e*_1_, *e*_2_ are disjoint, the edges *e*_3_, *e*_4_, *B*_1_, *B*_2_ are disjoint. Then, $\nabla \left(B_{i}\right)\;\left(i=1,2\right)$ forms a cyclical 4-edge-cut (see Figure 6F), by Lemma 2.3, $G≅Q_{l}\left(l\geq 1\right)$ .

Since $G_{0}=\left(X_{0},Y_{0}\right)$ is bipartite, it should be noted that each edge *e*_*i*_ of *E*_0_ is in these eight triangles and connects two vertices in *Y*_0_; thus, the edges of $E\left(G\right)-E\left(B_{1}\right)-E\left(B_{2}\right)$ are *X*_0_ *Y*_0_ − edges and *G* − *B*_1_ − *B*_2_ has only quadrangles (see Figure 6G). Moreover, by Lemma 2.3, we can know *G* − *B*_1_ − *B*_2_ consists of $l-2\;\left(l\geq 2\right)$ layers of quadrangles (each layer is made up of four quadrangles). Thus, we have $G\in H_{1}$ .

**Subcase 2.2:** $G\left[E_{0}\right]$ has exactly three components.

**FIGURE 7:**
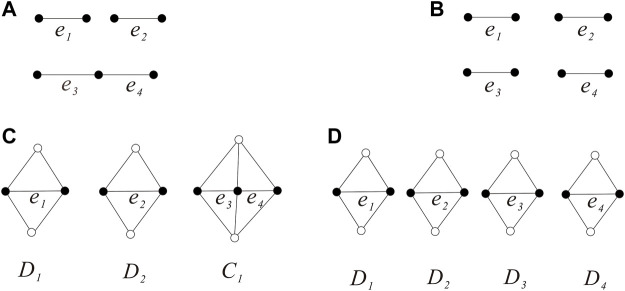
$G\left[E_{0}\right]$ has three components **(A,C)** or four components **(B,D)**.

Then, both of the two components of $G\left[E_{0}\right]$ are *K*_2_, and one component is *K*_1,2_ (see Figure 7A). Without loss of generality, we suppose the component *K*_1,2_ is induced by the edges *e*_3_, *e*_4_. Then, the two diamonds *D*_3_, *D*_4_ are adjacent, and *D*_1_, *D*_2_ are disjoint. Set *C*_1_ = *D*_3_ ∪ *D*_4_ (see Figure 7C).

Then, due to Lemma 2.3 and Lemma 3.1, $\nabla \left(C_{1}\right)$ forms a cyclical 4-edge-cut, thus, *G≅Q*_*s*_, where *Q*_*s*_ is the tubular {(3, 4), 4}-fullerene as shown in Figure 2, which means each of the two caps of *Q*_*s*_ must contain two adjacent diamonds, contradicts that *D*_1_, *D*_2_ are disjoint.

**Subcase 2.3:** $G\left[E_{0}\right]$ has four components.

Then, the four diagonal edges *e*_1_, *e*_2_, *e*_3_, and *e*_4_ are disjoint (see Figure 7B), that is, the four diamonds *D*_1_, *D*_2_, *D*_3_, and *D*_4_ cannot intersect at the diagonal edges. We have the four diamonds *D*_1_, *D*_2_, *D*_3_, and *D*_4_ as shown in Figure 7D. Then, $G\in H_{2}$ .

So far, we have completed the proof of Theorem 3.3.

Inspired by Theorem 3.3, we immediately get the following theorems.

Theorem 3.4 *Let* *G* *be a* {(3, 4), 4}*-fullerene, if* $G\in H_{1}$ *, then* $ak\left(G\right)=4$ *.*

Proof Let $G\in H_{1}$ , that is, $G≅Q_{n}\;\left(n\geq 0\right)$ . By Theorem 3.2 and the definition of the anti-Kekulé number, we only need to find an anti-Kekulé set *E*_0_ of *G* such that $\left|E_{0}\right|=4$ .

**FIGURE 8:**
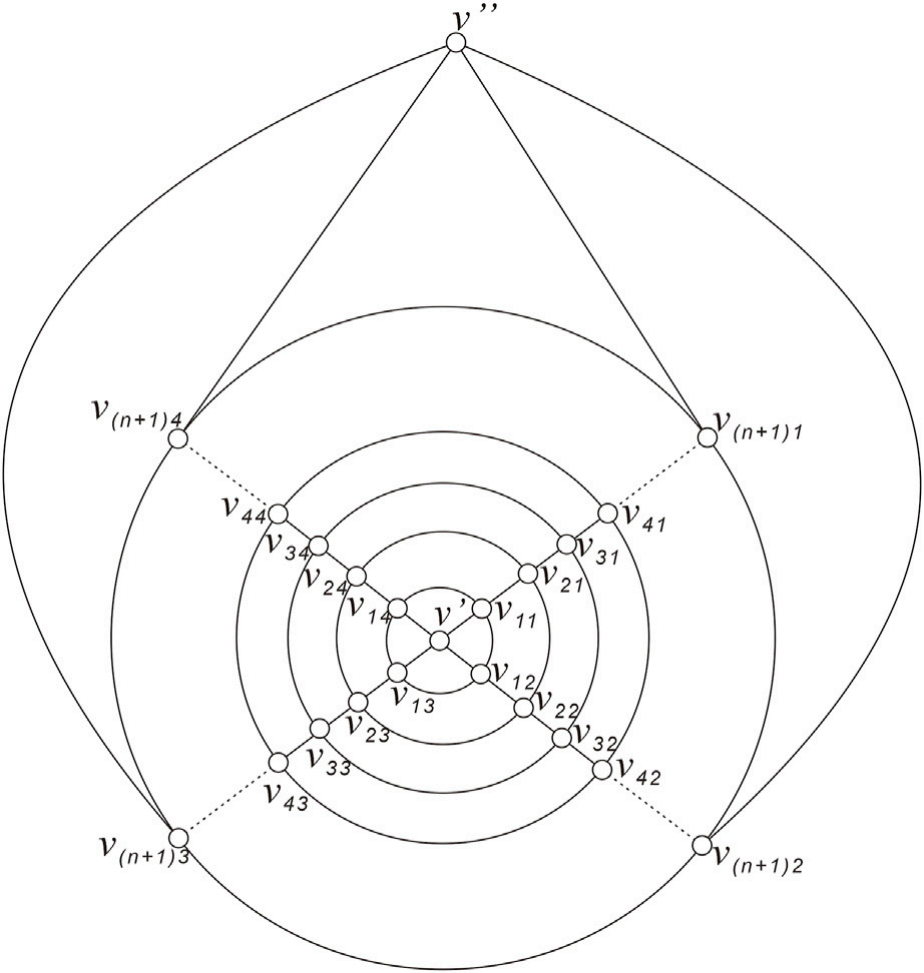
{(3,4),4}-Fullerenes *Q*_*n*_.

For convenience, let the plane embedding graph of *Q*_*n*_ as shown in Figure 8. *Q*_*n*_ consist of *n* + 1 concentric rings with four vertices on each ring and two vertices on two caps; these *n* + 1 concentric rings are recorded as *R*_1_, *R*_2_, *R*_3_, …, *R*_*n*+1_ from the inside to the outside. Next, the vertices of *Q*_*n*_ are labeled as follows: the vertices shared by the four triangles on the two caps are represented by *v*′, *v* ^″^, respectively, and the vertices on $R_{i}\;\left(i=1,2,3,\ldots ,n+1\right)$ are recorded as *v*_*i*1_, *v*_*i*2_, *v*_*i*3_, and *v*_*i*4_ (*i* = 1, 2, 3, …, *n* + 1) in a clockwise direction such that *v*_*i*1_, *v*_*i*3_ (*v*_*i*2_,, and *v*_*i*4_) are on the same line (see Figure 8 the labeling of *Q*_*n*_).

Next, we will prove Theorem 3.4 in two cases.

**Case 1:** *n* is an odd number.

**FIGURE 9:**
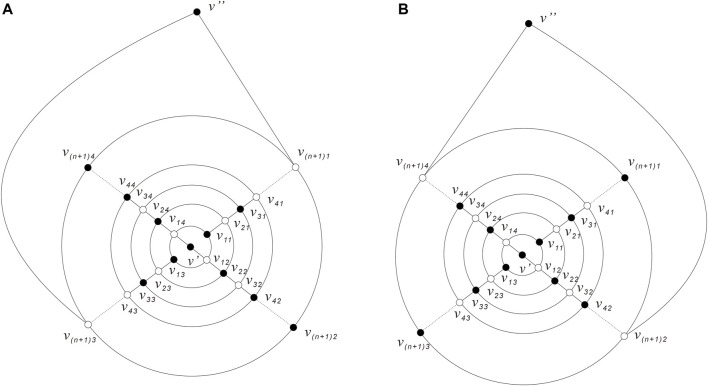
Graph *G* − *E*_0_; *n* is an odd number **(A)**, and *n* is an even number **(B)**.

Let $E_{0}=\left\{v^{\prime }v_{11},v^{\prime }v_{13},v^{\prime \prime }v_{n+1,2},v^{\prime \prime }v_{n+1,4}\right\}$ (see Figure 9A), and set *G*_1_ = *G* − *E*_0_. Then, *E*_0_ is not a cyclically 4-edge-cut of *G* by Lemma 2.3. Moreover, *E*_0_ is not a trivial 4-edge-cut as the four edges in *E*_0_ are not incident with a common vertex. That is, *G*_1_ is connected.

Then, we prove that *G*_1_ = *G* − *E*_0_ has no perfect matching, and there are only quadrangular faces in *G*_1_, so, *G*_1_ is bipartite. We color the vertices of *G*_1_ with black and white such that adjacent vertices in *G*_1_ are assigned two distinct colors (see Figure 9A). Let *M*_0_ denote the set of white vertices and *N*_0_ denote the set of black vertices, then $G_{1}=G_{1}\left(M_{0},N_{0}\right)$ , $\left|M_{0}\right|=2n+2$ , $\left|N_{0}\right|=2n+4$ . In accordance with Theorem 2.4, there exist $M_{0}\subseteq V\left(G_{1}\right)$ such that $o\left(G_{1}-M_{0}\right)=\left|N_{0}\right|=2n+4>\left|M_{0}\right|=2n+2$ , so *G*_1_ has no perfect matching.

**Case 2:** *n* is an even number.

Let $E_{0}=\left\{v^{\prime }v_{11},v^{\prime }v_{13},v^{\prime \prime }v_{n+1,1},v^{\prime \prime }v_{n+1,3}\right\}$ (see Figure 9B), and set *G*_2_ = *G* − *E*_0_. Also, *G*_2_ is connected.

There are only quadrangular faces in *G*_2_; so, *G*_2_ is also bipartite with one bipartition 2*n* + 2 vertices and the other bipartition 2*n* + 4 vertices, which means *G*_2_ has no perfect matching.

Therefore, we find the anti-Kekulé set *E*_0_ of *G* with $\left|E_{0}\right|=4$ , which means $ak\left(G\right)=4$ , if $G\in H_{1}$ .

Due to Theorem 3.4, if $G\in H_{1}$ , then $ak\left(G\right)=4$ . However, the anti-Kekulé number of *G* can be 4 or 5 if $G\in H_{2}$ . Next, we use a method to judge whether the anti-Kekulé number of *G* can be 4 or 5 when $G\in H_{2}$ . Before we give some definitions of *G* if $G\in H_{2}$ . Let $G\in H_{2}$ , the four diamonds of *G* be *D*_1_, *D*_2_, *D*_3_, and *D*_4_ and the four diagonal edges be *e*_1_, *e*_2_, *e*_3_, and *e*_4_ such that $e_{i}\in E\left(D_{i}\right)$ , *i* = 1, 2, 3, 4. Set $E_{0}=\left\{e_{1},e_{2},e_{3},e_{4}\right\}$ and *e*_1_ = *v*_1_ *v*_2_, *e*_2_ = *v*_3_ *v*_4_, *e*_3_ = *v*_5_ *v*_6_, and *e*_4_ = *v*_7_ *v*_8_. The eight vertices of the four diagonal edges are called eight *stars*, and their union is denoted by $V_{0}=\overset{8}{\underset{i=1}{⋃}}v_{i}$ .

Set *G*_0_ = *G* − *E*_0_. Then, *G*_0_ is bipartite, without loss of generality, we supposed the bipartitions of G_0_ were *V*_1_, *V*_2_. Then, by the proof of Theorem 3.3, we can know if $ak\left(G\right)=4$ , then *V*_0_ ⊂ *V*_1_ or *V*_0_ ⊂ *V*_2_, which means $ak\left(G\right)=5$ when *V*_0_⊄*V*_1_ and *V*_0_⊄*V*_2_. Thus, we have the following theorem.

Theorem 3.5 *Let* *G* *be a* {(3, 4), 4}*-fullerene,* $G\in H_{2}$ *, if* *V*_0_ ⊂ *V*_1_ *or* *V*_0_ ⊂ *V*_2_ *, then* $ak\left(G\right)=4$ *, otherwise,* $ak\left(G\right)=5$ *.*

Proof By Theorem 3.2, we only need to show if *V*_0_ ⊂ *V*_1_ or *V*_0_ ⊂ *V*_2_, then $ak\left(G\right)=4$ . Without loss of generality, suppose *V*_0_ ⊂ *V*_1_. Then, $G\left[V_{1}\right]$ consists of the four edges *e*_1_, *e*_2_, *e*_3_, and *e*_4_ and some singleton vertices. Since the four edges *e*_1_, *e*_2_, *e*_3_, and *e*_4_ cannot be incident with a common vertex, *E*_0_ is not a trivial 4-edge-cut. However, *E*_0_ also cannot be a cyclical 4-edge-cut by Lemma 2.3, as *e*_*i*_ belongs to the intersection of two triangular faces for *i* = 1, 2, 3, 4. Thus, *G*_0_ = *G* − *E*_0_ is connected.

On the other hand, by the degree-sum formula $4\left|V_{2}\right|=4\left|V_{1}\right|-8$ , which means $\left|V_{1}\right|\neq \left|V_{2}\right|$ . Thus, *G*_0_ cannot have perfect matchings by Theorem 2.4. So, we find the anti-Kekulé set *E*_0_ with $\left|E_{0}\right|=4$ . Immediately, we have $ak\left(G\right)=4$ . Otherwise, by Theorem 3.2, $ak\left(G\right)=5$ .

By Theorem 3.5, for a {(3, 4), 4}-fullerene *G* with $G\in H_{2}$ , we can give the method to judge the anti-Kekulé number of graph *G* is 4 or 5 as follows:

**Step 1:** Delete the four diagonal edges *e*_1_, *e*_2_, *e*_3_, and *e*_4_.
**Step 2:** Color the vertices of $G_{0}=G-\left\{e_{1},e_{2},e_{3},e_{4}\right\}$ with black and white.
**Step 3:** If we find the eight stars are in the same color, then $ak\left(G\right)=4$ , otherwise, $ak\left(G\right)=5$ .

## 4 Conclusion

In this paper, we have obtained the scope of the anti-Kekulé number of {(3, 4), 4}-fullerenes in Theorem 3.2; at the same time, we characterized {(3, 4), 4}-fullerenes with anti-Kekulé number 4 in Theorem 3.3, which includes two kinds of graphs $H_{1},H_{2}$ .

As a consequence, we proved that if $G\in H_{1}$ , then $ak\left(G\right)=4$ . Interestingly, by the proof of Theorem 3.3, we found the {(3, 4), 4}-fullerene *G* belongs to $H_{2}$ , but the anti-Kekulé number of *G* is not always 4; therefore, at the end of this paper, we gave a condition for judging whether the anti-Kekulé number of graph *G* is 4 or 5.

## Data Availability

The original contributions presented in the study are included in the article/Supplementary Material, further inquiries can be directed to the corresponding author.
